# The Intersection of Older Students and Disability: How Age-Friendly Universities Can Boost Visibility and Accessibility

**DOI:** 10.1093/geroni/igab046.2016

**Published:** 2021-12-17

**Authors:** Cassandra Barragan, Sarah Walsh

**Affiliations:** 1 Eastern Michigan University, Ypsilanti, Michigan, United States; 2 Eastern Michigan University, Eastern Michigan University, Michigan, United States

## Abstract

The needs of older learners differ from traditional students, and many services and resources available at higher education institutions are geared towards students aged 18-25 (Silverstein, Choi, & Bulot, 2001). Age Friendly University (AFU) principles highlight the need to consider older learners at a university. Older learners face various barriers to education including balancing schoolwork with responsibilities and accessibility of campus resources (Silverstein et al., 2001). This study examined how an AFU designated university is working to better understand their older students. Methods: A web-based pilot survey of older learners (N=248) asked all students ages 40 and older a series of questions regarding motivation to attend school, barriers and supports, campus environment, and connection with AFU principles. Analysis: A t-test analysis explored differences in motivation, barriers and challenges, and connection to campus between students who identified as having a disability and those who did not. Findings: We found there were significant differences between the groups in how health impacted their education (p=.001), being able to physically access campus (p=.014), the availability of online classes (p=.047), and the hours of operation of student support services (p=.045). There were also differences between groups in how connected they felt to campus based on feeling welcomed by faculty (p = .033) and feeling satisfied with their level of engagement at the university (p = .002). Discussion: Our results demonstrate the need to fully engage older learners with a disability as part of diversity and inclusion efforts to facilitate connection to the campus community.

